# Supplementary material for: Risk of metabolic syndrome in patients with lichen planus: A systematic review and meta-analysis
Source: PLoS One. 2020 Aug 21;15(8):e0238005. doi: 10.1371/journal.pone.0238005 (PMC7444576; doi:10.1371/journal.pone.0238005)
Supplement: S1 Table — (DOCX) [file pone.0238005.s002.docx]

**S1 Table. Search strategy**

| **Databases** | **Search terms** | **Date** |
| --- | --- | --- |
| PubMed | (metabolic syndrome OR syndrome X OR insulin resistance syndrome OR metabolic syndrome X OR Reaven syndrome) AND (lichen planus OR lesion planus)  Search option: All fields | 16 July 2020 |
| Cochrane library | (metabolic syndrome OR syndrome X OR insulin resistance syndrome OR metabolic syndrome X OR Reaven syndrome) AND (lichen planus OR lesion planus)  Search option: All fields | 16 July 2020 |
| Web of Science | (metabolic syndrome OR syndrome X OR insulin resistance syndrome OR metabolic syndrome X OR Reaven syndrome) AND (lichen planus OR lesion planus)  Search option: All fields | 16 July 2020 |
| Embase | (metabolic syndrome OR syndrome X OR insulin resistance syndrome OR metabolic syndrome X OR Reaven syndrome) AND (lichen planus OR lesion planus)  Search option: All fields | 16 July 2020 |
