# Supplementary material for: Risk of metabolic syndrome in patients with lichen planus: A systematic review and meta-analysis
Source: PLoS One. 2020 Aug 21;15(8):e0238005. doi: 10.1371/journal.pone.0238005 (PMC7444576; doi:10.1371/journal.pone.0238005)
Supplement: Attachment — Submitted filename: Response to Reviewers.docx [file pone.0238005.s003.docx]

**Dear Beatrice Nardone:**

**Thank you very much for giving us an opportunity to revise our manuscript. We appreciate the editor and reviewers very much for their constructive comments and suggestions on our manuscript entitled** **“Risk of metabolic syndrome in patients with lichen planus: A systematic review and meta-analysis” (ID: PONE-D-20-09664).**

**We have studied reviewers’ comments carefully. According to the reviewers’ detailed suggestions, we have made a careful revision on the original manuscript. All revised portions are marked in red in the revised manuscript which we would like to submit for your kind consideration.**

**In addition, we would like to change the financial disclosure to: WX received a research grant(No. 2020364003) from Zhejiang Medical and Health Science and Technology Program, China (URL:** <http://www.msttp.com/>**).** **The funder had no role in study design, data collection and analysis, decision to publish, or preparation of the manuscript.**

**We look forward to hearing from you soon.**

**Best wishes,**

**Wenzhong Xiang**

**E-mail: xiangwenzhong@126.com**

**Dear Beatrice Nardone and reviewers:**

**Thank you for your letter and the reviewers’ comments on our manuscript entitled “Risk of metabolic syndrome in patients with lichen planus: A systematic review and meta-analysis” (ID: PONE-D-20-09664).** **Those comments are very helpful for revising and improving our paper, as well as the important guiding significance to other research. We have studied the comments carefully and made corrections which we hope meet with approval. The main corrections are in the manuscript and the responds to the reviewers’ comments are as follows (the replies are highlighted in blue).**

**Replies to the reviewers’ comments:**

**Reviewer #1:**

**1. Why did the authors did not differ between the various clinical forms of LP?**

**Response：That's an excellent question. Clinical forms of LP including linear LP, hypertrophic LP, oral LP, LP pigmentosus, generalized LP, lichen planopilaris, bullous LP and actinic LP. Emerging evidences suggest that risk of MS varies depending on the clinical types of LP. However, only 3 out of 12 included studies provided original data or assessed the association between MS and different clinical forms of LP. Moreover, the amount of data after classification is too small to draw any conclusions. It is a prospective direction for further research work with research value, and we are planning to take this as the next research direction.**

**2. What is new about the findings compared to previously published data since link between LP and MS hat been previously reported?**

**Response: Previous studies investigating the relationship between LP and MS were observational studies, and no meta-analysis in this area has yet been published. In this meta-analysis, we examined data on the relationship between LP and odds of MS by searching for studies published before July 16, 2020. In addition, we also conducted a comparison of three different diagnostic criteria of MS.**

**Reviewer #2:**

**1. what was the aim of the study, why authors have stated that it is the first study investigated the prevalence of MS in the general population?**

**Response：The aim of the study is to systematically evaluate the published literatures and determine a clinical relationship between MS and LP. Currently, no systematic review and meta-analysis in this area has yet been published; therefore, we stated that “this is the first systematic review and meta-analysis of studies that investigated the prevalence of MS in patients with LP compared to the general population.”**

**Reviewer #3:**

**1. Why were participants with hypertension, diabetes and dyslipidemia excluded if these characteristics are criteria for the diagnosis of metabolic syndrome?**

**Response：Firstly, we aim to determine whether patients with LP are more likely to develop MS when compared with the general population; therefore, we need untested LP patients and controls, who are not sure if they have had MS before. Besides, participants with known hypertension, diabetes and dyslipidemia often have a long history of medication, and we cannot rule out the possibility of drug-induced MS.**

**2. There are diseases that are associated with metabolic syndrome. Were patients with these diseases excluded?**

**Response：Yes, skin disease such as psoriasis, atopic dermatitis, and vitiligo that are associated with MS were excluded. The outpatients with skin diseases were mainly nevi, seborrheic keratosis, actinic keratosis, verruca vulgaris, or basal cell carcinoma. We have added these to the “Materials and methods--Exclusion criteria” and “Results--Study characteristics” section.**

**3. We’ve also made the relevant changes to the Inclusion criteria, Literature search, Results and Discussion accordingly.**

**In addition, we have made the following changes to the article:**

**1. All three reviewers made reference to awkward sentences and lack of clarity in the flow of the text. We’ve addressed this issue across and within each section, paragraph, and sentence.**

**2. We re-searched the literature prior to July 16, 2020 according to the search strategy, and added 1 recently published eligible literature (Mushtaq S, Dogra D, Dogra N, Shapiro J, Fatema K, Faizi N, et al. Cardiovascular and Metabolic Risk Assessment in Patients with Lichen Planus: A Tertiary Care Hospital-based Study from Northern India. Indian Dermatol Online J. 2020;11(2):158-66.).**

**3. We removed Medline from the databases because PubMed comprises citations for biomedical literature from MEDLINE. The number of results is different because the new PubMed, launched after 18 May 2020, has some changes in search syntax and search translations.**

**4.** **We reedited the article to ensure that it meets PLOS ONE's style requirements and assessed study quality based on the Newcastle-Ottawa Scale. Complete search strategy** **is shown in S2 Table.**

**Once again, thank you very much for your constructive comments and suggestions which would help us both in English and in depth to improve the quality of the paper.**

**We look forward to hearing from you soon.**

**Best wishes,**

**Wenzhong Xiang**

**E-mail: xiangwenzhong@126.com**
